# Metformin and risk of gingival/periodontal diseases in diabetes patients: A retrospective cohort study

**DOI:** 10.3389/fendo.2022.1036885

**Published:** 2022-10-05

**Authors:** Chin-Hsiao Tseng

**Affiliations:** ^1^ Department of Internal Medicine, National Taiwan University College of Medicine, Taipei, Taiwan; ^2^ Division of Endocrinology and Metabolism, Department of Internal Medicine, National Taiwan University Hospital, Taipei, Taiwan

**Keywords:** gingival and periodontal diseases, metformin, pharmacoepidemiology, Taiwan, diabetes mellitus

## Abstract

**Aim:**

To compare the risk of gingival and periodontal diseases (GPD) between ever users and never users of metformin in patients with type 2 diabetes mellitus.

**Methods:**

The Taiwan’s National Health Insurance database was used to enroll 423,949 patients with new onset diabetes mellitus from 1999 to 2005. After excluding ineligible patients, 60,309 ever users and 5578 never users were followed up for the incidence of GPD from January 1, 2006 until December 31, 2011. Propensity score-weighted hazard ratios were estimated by Cox regression.

**Results:**

GPD was newly diagnosed in 18,528 ever users (incidence: 7746.51 per 100,000 person-years) and 2283 never users (incidence: 12158.59 per 100,000 person-years). The hazard ratio that compared ever users to never users was 0.627 (95% confidence interval: 0.600-0.655). When metformin use was categorized by tertiles of cumulative duration and cumulative dose, the risk significantly reduced in a dose-response pattern when the cumulative duration reached approximately 2 years or the cumulative dose reached 670 grams. Analyses on the tertiles of defined daily dose of metformin showed that the reduction of GPD risk could be seen in all three subgroups but the benefit would be greater when the daily dose increased.

**Conclusion:**

Long-term use of metformin is associated with a significantly reduced risk of GPD.

## Introduction

Gingivitis and periodontitis are very common diseases in the oral cavity (1). Gingivitis refers to reversible inflammation of the gingiva and periodontitis shows irreversible destruction of the supporting tissues around the teeth with potential risk of bone loss and teeth loss (2). According to the 2009-2012 US National Health and Nutrition Examination Survey, periodontitis was diagnosed in approximately 46% of adults aged 30 years or older (3). A 2015-2016 survey conducted in 10,281 adults aged 18 years or older in Taiwan showed a prevalence rate of GPD of 80.5% (4). Risk factors of GPD include aging, diabetes mellitus, human immunodeficiency virus infection, smoking, poor oral hygiene, post-menopause (estrogen deficiency), inflammatory bowel disease and osteoporosis etc. (2, 5, 6).

Diabetes patients have an increased risk of GPD because of the high infection rate, high oxidative stress, immune dysfunction and pro-inflammatory status associated with hyperglycemia and the metabolic and hemodynamic disturbances (2). Metformin, an old oral antidiabetic drug, is currently used as the first-line treatment in patients with type 2 diabetes mellitus (T2DM). Over the world, >150 million diabetes patients are being prescribed metformin (7). Besides a glucose-lowering effect, metformin shows multiple pleiotropic benefits including endothelial protection, anti-atherosclerosis, anti-neoplasm, anti-inflammation, anti-microbia, immune modulation, anti-aging and pro-osteogenesis (8–12). In our previous observational studies conducted in Taiwan, we did show that metformin users, when compared to non-users, have a lower risk of various types of cancer including oral cancer (13), endometrial cancer (14), breast cancer (15), prostate cancer (16, 17), kidney cancer (18), bladder cancer (19), liver cancer (20), pancreatic cancer (21) and malignant brain tumors (22). Additionally, metformin use has also been observed to be associated with a lower risk of non-cancerous diseases such as *Helicobacter pylori* infection (23), tuberculosis infection (24), dementia (25, 26), inflammatory bowel disease (27), diverticula of intestine (28), hemorrhoid (29), varicose veins (30), osteoporosis/vertebral fracture (31), hypertension (32), atrial fibrillation (33) and heart failure (34). These findings support metformin’s anti-neoplastic, anti-inflammatory, anti-microbial and pro-osteogenic actions in humans.

Metformin distributes to various tissues including the salivary gland, oral mucosa, tongue, bone marrow, and the gastrointestinal tracts of stomach, small intestine, colon and appendix (35, 36). An early randomized controlled trial in humans suggested a potential usefulness of metformin in the treatment of GPD by locally delivering metformin into the periodontal pockets (37). Some later randomized controlled trials supported a potential usefulness of 1% metformin gel for the treatment of chronic periodontitis (38–40).

Although a handful of previous research focused on the usefulness of metformin for the treatment of periodontitis, whether metformin may render a protective effect on the development of GPD has not yet been studied. In this retrospective cohort study, we investigated the risk of GPD in patients with T2DM with regards to the exposure to metformin.

## Materials and methods

### Enrollment of study subjects

Taiwan started to implement a nationwide and compulsory healthcare system, the National Health Insurance (NHI), since March 1, 1995. This healthcare system covers > 99% of the Taiwan’s population. Across the country, all in-hospitals and more than 93% of the medical settings are contracted with the Bureau of the NHI to provide medical care to the insurants. For reimbursement, the Bureau of the NHI requests the submission of computerized medical records including the diagnoses of diseases, prescriptions of drugs and clinical procedures performed. Academic researchers can use the database for clinical investigation if their proposals are approved after institutional ethics review. This retrospective cohort study was conducted according to the local regulations after review and approval (approval number: NHIRD-102-175) by the Research Ethics Committee of the National Health Research Institutes. For the protection of privacy, personal information had been de-identified in the database before it was released for analyses. Therefore, according to local regulations, informed consent was not required because there was no way to contact the individuals.

During the whole study period, the coding system for disease diagnoses in the database was the International Classification of Diseases, Ninth Revision, Clinical Modification (ICD-9-CM). Accordingly, diabetes mellitus was coded by 250.XX and GPD by 523.

**Figure 1:**
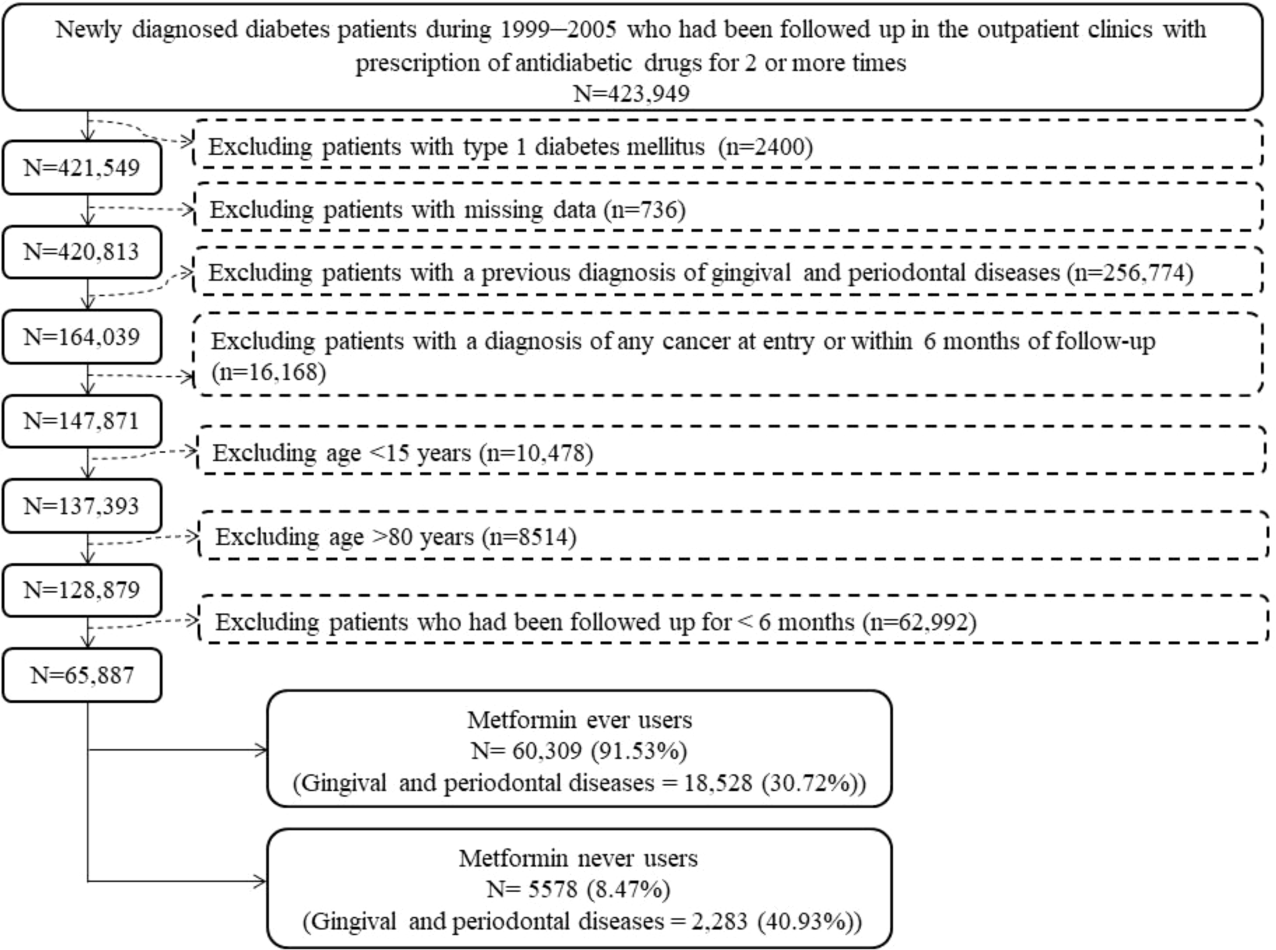
The procedures in the flowchart followed in creating a cohort of ever users and never users of metformin for analyses.

**Figure 1** shows the flowchart followed to create a cohort of ever users and never users of metformin used for analyses. At first, 423,949 patients were identified. These patients should have had a new diagnosis of diabetes mellitus in 1999-2005 and they should also have received prescriptions of antidiabetic drugs for at least two times at the outpatient clinics. Ineligible patients were then excluded step-by-step. As a result, we finally enrolled 65,887 patients (60,309 ever users and 5578 never users) into the study.

### Potential confounders

**Table 1** shows the variables treated as potential confounders. The ICD-9-CM codes of the disease diagnoses have been reported previously (28). They were selected because of a potential correlation with the exposure (i.e., metformin) or the outcome (i.e., GPD) or because these diagnoses might have potential detrimental effects on the patients’ life expectancy that might have led to a biased calculation of the incidence. Diseases that require the use of antibiotics, steroids and anti-inflammatory drugs for a long time were especially considered because the risk of GPD might have been affected by the use of these drugs. According to the Bureau of the NHI, four classes of occupation were defined: (I) civil servants, teachers, employees of governmental or private businesses, professionals and technicians; (II) people without a specific employer, self-employed people and seamen; (III) farmers and fishermen; and (IV) low-income families supported by social welfare and veterans. Five categories of living regions were classified according to geographical distribution: Taipei, Northern, Central, Southern, and Kao-Ping/Eastern.

**Table 1:** Characteristics in never users and ever users of metformin.

	Never users		Ever users		
Variables	(n = 5578)		(n = 60309)		Standardized difference
	n	%	n	%	
**Basic data**					
Age* (years)	61.58	11.93	59.42	11.68	-19.74
Sex (men)	2994	53.68	31561	52.33	-2.65
Occupation					
I	1688	30.26	18756	31.10	
II	1001	17.95	12250	20.31	6.27
III	1664	29.83	17923	29.72	0.07
IV	1225	21.96	11380	18.87	-8.16
Living region					
Taipei	1468	26.32	15464	25.64	
Northern	573	10.27	7634	12.66	7.87
Central	855	15.33	9402	15.59	0.61
Southern	1266	22.70	13154	21.81	-1.85
Kao-Ping and Eastern	1416	25.39	14655	24.30	-2.18
**Major comorbidities of diabetes**					
Hypertension	4265	76.46	43725	72.50	-9.89
Dyslipidemia	3006	53.89	37687	62.49	18.62
Obesity	101	1.81	1939	3.22	9.06
**Diabetes-related complications**					
Nephropathy	1347	24.15	9713	16.11	-23.00
Eye diseases	509	9.13	8611	14.28	16.22
Diabetic polyneuropathy	563	10.09	10437	17.31	21.20
Stroke	1552	27.82	13637	22.61	-13.89
Ischemic heart disease	2027	36.34	20412	33.85	-5.90
Peripheral arterial disease	853	15.29	10181	16.88	3.65
**Antidiabetic drugs**					
Insulin	504	9.04	1479	2.45	-30.59
Sulfonylurea	4244	76.08	41855	69.40	-9.28
Meglitinide	452	8.10	2202	3.65	-20.24
Acarbose	493	8.84	2969	4.92	-14.55
Rosiglitazone	161	2.89	2543	4.22	8.00
Pioglitazone	103	1.85	1368	2.27	3.61
**Drugs commonly used by diabetes patients**					
Angiotensin converting enzyme inhibitors/ angiotensin receptor blockers	3407	61.08	36385	60.33	-1.79
Calcium channel blockers	3294	59.05	32162	53.33	-12.32
Statins	1955	35.05	24629	40.84	12.95
Fibrates	1380	24.74	17833	29.57	11.36
Aspirin	2630	47.15	28909	47.93	0.91
**Drugs that may affect the outcome**					
Non-steroidal anti-inflammatory drugs	1902	34.10	18544	30.75	-7.90
Selective serotonin re-uptake inhibitors	319	5.72	3057	5.07	-3.32
Opioid analgesics	859	15.40	8409	13.94	-5.10
Immunosuppressants	261	4.68	2045	3.39	-8.10
**Common comorbidities that may affect the exposure/outcome**					
Chronic obstructive pulmonary disease	2129	38.17	22300	36.98	-3.38
Tobacco abuse	79	1.42	911	1.51	1.19
Alcohol-related diagnoses	347	6.22	3253	5.39	-4.50
Heart failure	968	17.35	7838	13.00	-13.87
Parkinson’s disease	150	2.69	1039	1.72	-7.59
Dementia	297	5.32	2476	4.11	-6.75
Head injury	71	1.27	706	1.17	-1.29
Valvular heart disease	426	7.64	3557	5.90	-8.31
Pneumonia	625	11.20	5459	9.05	-9.17
Osteoporosis	942	16.89	9404	15.59	-4.25
Arthropathies and related disorders	3530	63.28	38364	63.61	0.58
Psoriasis and similar disorders	90	1.61	1142	1.89	2.13
Dorsopathies	3473	62.26	38972	64.62	5.19
Liver cirrhosis	279	5.00	1962	3.25	-10.38
Other chronic non-alcoholic liver diseases	349	6.26	4231	7.02	3.31
Hepatitis B virus infection	62	1.11	646	1.07	-0.54
Hepatitis C virus infection	202	3.62	1957	3.24	-2.26
Human immunodeficiency virus infection	3	0.05	30	0.05	-0.57
Organ transplantation	17	0.30	66	0.11	-5.62
*Helicobacter pylori* infection	23	0.41	226	0.37	-0.54
Peptic ulcer site unspecified	1808	32.41	19203	31.84	-1.99
Appendicitis	97	1.74	929	1.54	-2.38
Noninfective enteritis and colitis	2275	40.79	25232	41.84	1.82
Irritable bowel syndrome	566	10.15	5720	9.48	-2.58
Anal fissure/fistula	93	1.67	1102	1.83	0.79
Abscess of anal/rectal regions	68	1.22	987	1.64	3.68
Episodic mood disorders	208	3.73	2018	3.35	-2.75
Depressive disorder	119	2.13	1261	2.09	-0.37
Drug dependence	31	0.56	176	0.29	-4.61

*Age is denoted as mean and standard deviation.

The definitions of occupation can be seen in “Materials and Methods”.

The accuracy of the ICD-9-CM codes labelled in the NHI database have been previously studied (41, 42). When ICD-9-CM codes 250.XX were used for diabetes mellitus, the sensitivity and positive predictive value were 90.9% and 90.2%, respectively (41). Moderate to substantial agreements between claim data and medical records were found and their Kappa values ranged from 0.55 to 0.86 (42).

### Statistical analyses

We used the SAS statistical software (SAS Institute, Cary, NC), version 9.4, for statistical analyses. A *P* value < 0.05 was considered as an indicator of statistical significance.

Standardized difference was calculated according to Austin and Stuart for each variable (43). A variable was considered to exert potential confounding if its standardized difference was > 10%.

Prescriptions in the database were used to calculate the cumulative duration (expressed in months) and cumulative dose (expressed in mg) of metformin therapy and their tertile cutoffs were used to assess a dose-response relationship (14). Additionally, defined daily dose (DDD) of metformin was used to investigate whether the risk might differ with regards to the daily dose of metformin. One unit of DDD of metformin is equal to 2 grams (14). Incidence density was calculated for different subgroups according to the exposure to metformin, i.e., never users, ever users and ever users stratified by the tertiles of cumulative duration, cumulative dose and DDD of metformin therapy. January 1, 2006 was set as the starting date of follow-up. The incidence numerator was the number of new GPD cases that were identified during follow-up. The incidence denominator was the follow-up time expressed in person-years, which was calculated from January 1, 2006 until December 31, 2011 when whichever of the following events occurred first: a new GPD diagnosis, death or the last reimbursement record available.

Cumulative incidence functions for GPD were plotted with regards to metformin exposure and Gray’s test was used to test the difference between ever and never users.

Propensity score (PS) was created by logistic regression that included the date of entry and all the variables listed in **Table 1** as independent variables. To reduce the potential confounding from the differences in characteristics between ever and never users, PS-weighted hazard ratios were derived from Cox regression incorporated with the inverse probability of treatment weighting (IPTW) (44). In the main analyses, we estimated hazard ratios that compared ever users to never users, and compared each tertile of the cumulative duration, cumulative dose and DDD to never users.

### Sensitivity analyses

The hazard ratios for ever users versus never users in the following restricted subgroups were then conducted as sensitivity analyses to examine the consistency of the findings: I. Censoring patients at a time when the last prescription had elapsed a period of >4 months; II. Excluding patients who had been previously treated by other antidiabetic drugs when metformin was first prescribed (This exclusion precluded the possible carry-over effect exerted by other antidiabetic drugs.); III. Excluding patients who had been followed up for a duration of shorter than 12 months; IV. Excluding patients who had used metformin for <12 months; V. Analysis was conducted by enrolling patients during 1999-2002; VI. Analysis was conducted by enrolling patients during 2003-2005; VII. Excluding patients whose two consecutive prescriptions of metformin spanning >4 months (The NHI allows a drug prescription of <3 months at each time, therefore, patients who had a delayed refill might have been irregularly followed.); VIII. Patients who had been prescribed incretin-based therapies during follow-up were excluded (The NHI did not reimburse the first incretin-based therapy until after 2009.); IX. Analysis restricted to male patients; and X. Analysis restricted to female patients.

## Results

The characteristics of patients with regards to metformin exposure are shown in **Table 1**. Variables that had a value of standardized difference > 10% were age, dyslipidemia, nephropathy, eye diseases, diabetic polyneuropathy, stroke, insulin, meglitinide, acarbose, calcium channel blockers, statins, fibrates, heart failure and liver cirrhosis. The imbalance in some potential confounders justified the use of the IPTW method to estimate hazard ratios weighted for PS, as recommended by Austin (44).

**Figure 2:**
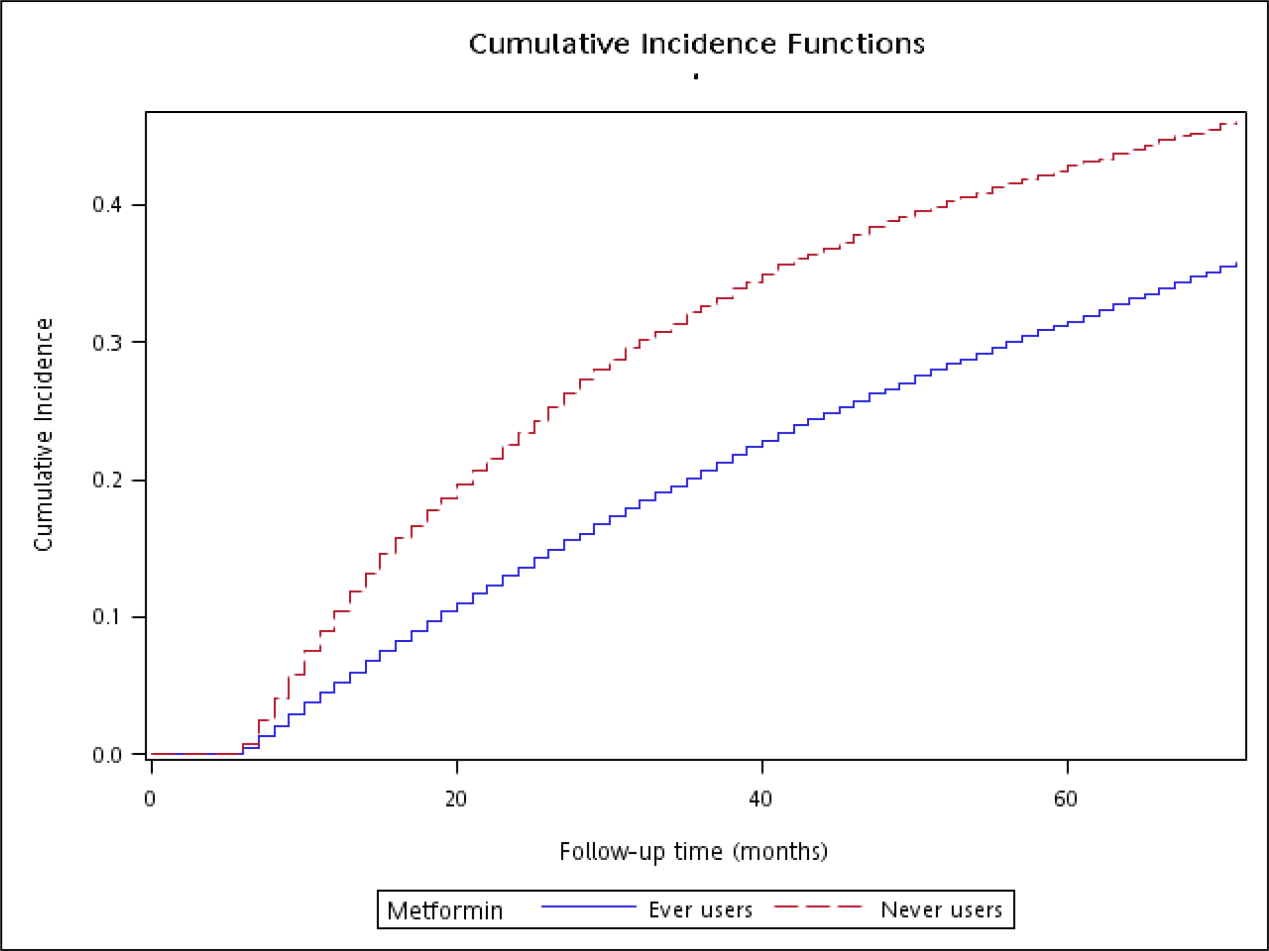
The cumulative incidence function for gingival and periodontal diseases with regards to metformin exposure (Gray’s test *P* < 0.01).

The cumulative incidence functions are shown in **Figure 2**. A significantly lower risk among ever users was observed when compared to never users (*P* < 0.01, Gray’s test).

The main analyses on the incidences and the hazard ratios of GPD according to metformin exposure are shown in **Table 2**. The incidence of GPD after a median follow-up of 3.15 years in never users was 12158.59 per 100,000 person-years. In ever users, after a median follow-up of 4.38 years, the incidence was 7746.51 per 100,000 person-years. Overall, a significant risk reduction of 37% was observed among ever users. The tertile analyses suggested that the risk significantly reduced in a dose-response pattern when the cumulative duration reached approximately 2 years or the cumulative dose reached 670 g despite a significantly higher risk being observed in the respective first tertiles. The tertile analysis on DDD suggested that the benefit could be observed in any of the DDD and the benefit would be greater when the daily doses increased accordingly.

**Table 2:** Incidences of gingival and periodontal diseases with regards to metformin exposure and hazard ratios comparing metformin exposure to never users.

Metformin use	Incident case number	Cases followed	Person-years	Incidence rate (per 100,000 person-years)	Hazard ratio	95% Confidence interval	*P* value
Never users	2283	5578	18776.84	12158.59	1.000		
Ever users	18528	60309	239178.60	7746.51	0.627	(0.600-0.655)	<0.0001
**Tertiles of cumulative duration of metformin therapy (months)**							
Never users	2283	5578	18776.84	12158.59	1.000		
<22.40	7481	19821	55111.19	13574.38	1.099	(1.048-1.152)	<0.0001
22.40-52.20	6715	19971	78471.42	8557.26	0.682	(0.650-0.715)	<0.0001
>52.20	4332	20517	105595.99	4102.43	0.306	(0.291-0.322)	<0.0001
**Tertiles of cumulative dose of metformin therapy (grams)**							
Never users	2283	5578	18776.84	12158.59	1.000		
<669.65	7368	19901	56420.60	13059.06	1.054	(1.005-1.105)	0.0291
669.65-1779.00	6506	19895	79627.31	8170.56	0.652	(0.622-0.684)	<0.0001
>1779.00	4654	20513	103130.68	4512.72	0.342	(0.325-0.360)	<0.0001
**Tertiles of units of defined daily dose of metformin therapy**							
Never users	2283	5578	18776.84	12158.59	1.000		
<0.50	6206	19901	74696.24	8308.32	0.676	(0.644-0.709)	<0.0001
0.50-0.64	6111	19902	78478.66	7786.83	0.632	(0.603-0.664)	<0.0001
>0.64	6211	20506	86003.69	7221.78	0.581	(0.554-0.610)	<0.0001

The sensitivity analyses in **Table 3** consistently showed a lower risk of GPD in ever versus never users and the preventive effect of metformin could be similarly shown in men and in women.

**Table 3:** Sensitivity analyses.

Metformin use	Incident case number	Cases followed	Person-years	Incidence rate (per 100,000 person-years)	Hazard ratio	95% Confidence interval	*P* value
**1. Censoring patients when four months have elapsed since the last prescription**							
Never users	2283	5578	18776.84	12158.59	1.000		
Ever users	15790	60309	208089.89	7588.07	0.621	(0.595-0.649)	<0.0001
**2. Excluding patients who had received other antidiabetic drugs before the first dose of metformin was prescribed**							
Never users	2283	5578	18776.84	12158.59	1.000		
Ever users	8848	27253	108970.07	8119.66	0.658	(0.628-0.689)	<0.0001
**3. Excluding patients who had a duration of follow-up for less than twelve months**							
Never users	1759	4809	18203.60	9662.92	1.000		
Ever users	15614	55898	235888.29	6619.23	0.665	(0.633-0.698)	<0.0001
**4. Excluding patients who had been treated wtih metformin for less than twelve months**							
Never users	2283	5578	18776.84	12158.59	1.000		
Ever users	14417	49113	210129.60	6861.00	0.544	(0.521-0.569)	<0.0001
**5. Analysis restricted to patients enrolled from 1999 to 2002**							
Never users	868	2289	7683.94	11296.29	1.000		
Ever users	9552	31891	128568.76	7429.49	0.647	(0.603-0.693)	<0.0001
**6. Analysis restricted to patients enrolled from 2003 to 2005**							
Never users	1415	3289	11092.90	12755.91	1.000		
Ever users	8976	28418	110609.83	8115.01	0.626	(0.592-0.663)	<0.0001
**7. Excluding patients who had received two consecutive prescriptions of metformin spanning >4 months**							
Never users	2283	5578	18776.84	12158.59	1.000		
Ever users	5167	17840	68870.76	7502.46	0.611	(0.581-0.642)	<0.0001
**8. Excluding patients who had been prescribed incretin-based therapies during follow-up**							
Never users	2264	5394	17910.79	12640.42	1.000		
Ever users	17625	51530	195117.87	9033.00	0.704	(0.674-0.736)	<0.0001
**9. Analysis restricted to male patients**							
Never users	1216	2994	9904.39	12277.38	1.000		
Ever users	9726	31561	122890.33	7914.37	0.633	(0.597-0.672)	<0.0001
**10. Analysis restricted to female patients**							
Never users	1067	2584	8872.45	12026.00	1.000		
Ever users	8802	28748	116288.26	7569.12	0.620	(0.582-0.661)	<0.0001

## Discussion

### Main findings

This is the first population-based retrospective cohort study that showed a preventive role of metformin in the occurrence of GPD. The findings of an overall lower risk associated with metformin use were consistently observed in different analyses (**Tables 2**, **3**). Although a significantly higher risk could be seen in the first tertiles of cumulative duration and cumulative dose, a dose-response effect with a significantly lower risk in the second and third tertiles suggested a potential cause-effect relationship (**Table 2**). The lower risk could be seen in all subgroups of DDD, but a higher DDD seemed to provide a better protection (**Table 2**).

### Potential mechanisms

Although not yet completely researched, the glucose lowering effect and the anti-inflammatory, anti-microbial, and pro-osteogenic properties of metformin (8) might have explained the potential mechanisms of such a reduced risk of GPD associated with metformin use. Metformin may also influence the development of GPD by modifying oral and gut microbiota.

A recent study that used *in vitro* and *in vivo* diabetes models suggested that hyperglycemia and inflammation interacted to play an important role in the development of GPD (45). In the *in vivo* studies conducted in gingival epithelium and serum collected from controls and diabetes patients and mice, the burden of senescent cells in gingival epithelium and the secretion of senescence-associated secretory phenotype in the serum were significantly higher in diabetes patients and mice than in the controls (45). In the *in vitro* study, hyperglycemia induced inflammaging in human oral keratinocytes, which could be alleviated by inhibiting the activation of inflammasomes (45). Therefore, the inflammaging induced by hyperglycemia through inflammasome activation may destruct the gingival epithelia barrier function in diabetes patients, leading to the onset, development and progression of GPD. Metformin may modulate inflammation by ameliorating hyperglycemia and through an 5’ adenosine monophosphate-activated protein kinase-dependent modulation of the mammalian target of rapamycin and the signal transducer and activator of transcription 3 and 5 of T-cells (46). Moreover, another role which could be played by metformin is the effect on the body weight. It is well known that adipose tissue represents an endocrine organ able to produce several adipokines which, when increased, may upregulate the inflammation and predisposed to cardiovascular and kidney diseases (47). Metformin, by contrasting obesity, is able to reduce the excess of adipokines, thus reducing inflammation (48).

Metformin exerts antibacterial activity against pathogens linked to periodontitis such as *Porphyromonas gingivalis* and *Tannerella forsythia* (49). It also inhibits the expression of inflammatory cytokines such as interleukin-6, interleukin-1β and tumor necrosis factor alpha in human gingival fibroblasts activated by *Porphyromonas gingivalis* (50).

Metformin may induce the differentiation of osteoblasts resulting in bone formation (51). This counteracts the osteoclastogenic activity associated with GPD (6). Patients with osteoporosis are at risk of GPD (5) and our recent study did suggest a significantly reduced risk of osteoporosis/vertebral fracture associated with metformin use (31). Thus, metformin may prevent the bone loss related to periodontitis.

A recent human study showed that the composition of salivary microbiota might change while patients with T2DM were treated with antidiabetic drugs and the microbiota might vary by the use of metformin (52). Another study suggested that metformin treatment for at least 6 months with adequate glycemic control (hemoglobin A_1c_< 6.5%) in T2DM patients with periodontitis led to a resemblance of salivary microbiota to the pattern of healthy individuals (53). Therefore, though not yet extensively studied, the changes in salivary microbiota by metformin might contribute to a prevention in the pathogenesis of GPD.

There are interactions between oral and gut microbiota. Periodontal pathogens may affect intestinal barrier (54) and oral infection with *Porphyromonas gingivalis* was associated with a reduction of *Akkermansia muciniphila* in the gut (55). On the other hand, metformin treatment is known to increase the proliferation of *Akkermansia muciniphila* in the gut (56). Although intestinal butyrate produced from gut microbiota is beneficial to human health, oral butyrate-producing bacteria may promote the development of periodontitis, suggesting that butyrate may be a double-edged sword in the development of GPD (57). Periodontal pathogens like *Porphyromonas gingivalis* and *Fusobacterium nucleatum* are butyrate-producing in the oral cavity (57). This implies that the antibacterial effect of metformin on *Porphyromonas gingivalis* (49) may reduce the production of butyrate in the oral cavity. It would be interesting to explore whether the slightly but significantly higher risk of GPD in the first tertiles of cumulative duration and cumulative dose of metformin therapy observed in this study (**Table 2**) could be due to an increase of butyrate-producing bacteria in the oral cavity, which would then be counteracted by an increase in the proliferation of *Akkermansia muciniphila* in the gut (56) after a longer duration or a larger cumulative dose of metformin therapy (**Table 2**).

### Clinical implications

There are some clinical implications. First, metformin may provide an additional bonus of reducing the risk of GPD besides other pleiotropic benefits. Because GPD is very common, clinical and economical burdens of GPD can be much reduced by using a very inexpensive antidiabetic drug. As calculated from the data, the large absolute risk reduction of 10.2% (2283/5578 − 18528/60309 = 10.2%) and the small number needed to treat of 10 (calculated as the reciprocal of absolute risk reduction) indicated that the use of metformin to prevent GPD may be cost-effective.

Second, because GPD is associated with systemic inflammatory diseases such as T2DM, cardiovascular disease, rheumatoid arthritis, Alzheimer’s disease, autoimmune diseases and cancer (6), prevention of GPD is also expected to reduce the burden of many inflammatory diseases.

Third, because of the dose-response effect (**Table 2**) and the potential mechanisms independent of glycemic control, it is reasonable to recommend a continuation of metformin use in the absence of contraindications when other antidiabetic drugs are added for further improvement of hyperglycemia.

Fourth, the findings of this observational study give sufficient rationale to design large clinical trials to confirm the benefit of metformin in the prevention of GPD.

### Limitations

There are some potential limitations. First, we recognized that this is a retrospective cohort study and not a randomized clinical trial. Although the statistical analyses suggested an inverse association between metformin and GPD, this does not necessarily imply a causation (58).

Second, hyperglycemia is an important risk factor for the development of chronic complications of diabetes (59, 60) and GPD (45). However, we did not have biochemical information of glycemic control such as fasting blood glucose, postprandial blood glucose or hemoglobin A_1c_ for adjustment in the analyses. In secondary analyses, we analyzed the correlation between diabetic microangiopathies (retinopathy and nephropathy, respectively) and GPD and have found an inverse correlation between either retinopathy or nephropathy and GPD (*P* < 0.001). At first glance, this might seem to be conflicting to the concept of a link among glycemic control, diabetic microangiopathies and GPD. However, this inverse correlation might have been explained on the ground that the attending physicians of patients with diabetic microangiopathies at baseline might have especially advised a stricter control of blood glucose to their patients. At the same time, the patients having microangiopathies might have a greater motivation to control their blood glucose to a better level. Therefore, the better glycemic control among patients with pre-existing microangiopathy at baseline might have lowered the incidence of GPD during follow-up in the study. It should be noted that patients with GPD had been excluded at the start of follow-up. Therefore, the inverse correlation between diabetic microangiopathy at baseline and GPD diagnosed during follow-up should not be interpreted as a lack of importance of glycemic control on the development of GPD. More future studies are required to clarify the cause-effect relationship between hyperglycemia and GPD with the consideration of baseline biochemical profiles of blood glucose and/or hemoglobin A_1c_.

Third, GPD may also be prevented by maintaining oral hygiene and reducing pathogenic bacteria with tooth brushing, interdental cleaning, essential oil mouthwash, cessation of cigarette smoking and intake of probiotics and antioxidants (1). Because we did not have related information, the role of these unmeasured confounders should be considered in future studies.

Fourth, we would expect some misclassifications of disease diagnoses in the database. However, because the misclassifications were expected to be nondifferential, the hazard ratios might have only been biased toward the null.

Fifth, the median follow-up of 3.15 years in never users and 4.38 years in ever users might be too short for a long-term outcome. However, the consistency of the findings (**Tables 2**, **3**) and the dose-response effect (**Table 2**) implied a robustness of the results.

Sixth, we did not have clinical, histological and radiographical data for disease confirmation and assessment of disease severity.

### Strengths

This study may have some strengths. First, because the database and the sample size were large and the enrollment period from 1999 to 2005 was long, selection bias and lack of statistical power might have been avoided and the findings could be more readily applied to the general patients in the population.

Second, by using preexisting records, we could avoid self-reporting bias and recall bias. Furthermore, prevalent user bias could be prevented by including only new users of metformin (**Figure 1**).

Third, we aimed at reducing immortal time bias during the design of the study by more appropriate assignment of treatment status and less miscalculation of follow-up time. Because we retrieved all longitudinal information, misclassification of treatment status was not likely and the cumulative duration, cumulative dose and DDD could be more accurately calculated. To assure a diagnosis of diabetes mellitus, we included only patients who had prescriptions of antidiabetic drugs for at least two times (**Figure 1**). We deliberately excluded two periods of potential immortal time in the calculation of person-years of follow-up: 1) the period between the diagnosis of diabetes mellitus and the first prescription of antidiabetic drugs; and 2) patients who had a short follow-up duration of <6 months. In Taiwan’s NHI healthcare system, the immortal time between hospital discharge and drug dispense is not a problem because all discharge prescriptions can be dispensed at the hospital on the day of discharge.

Fourth, in some countries disease detection rate is much affected by socioeconomic status. However, this bias is not a big problem in Taiwan because of the low cost-sharing in our healthcare system. Furthermore, in patients with low income and veterans and when prescriptions are refilled for chronic diseases, much expense can actually be waived.

## Conclusions

This is the first population-based cohort study that used a nationwide healthcare insurance database of Taiwan to demonstrate a preventive role of metformin in GPD development after a long cumulative duration of approximately 2 years or after a large cumulative dose of approximately 670 grams. Because of some inherent limitations associated with observational study design, additional studies or clinical trials are warranted to confirm the findings. The recommendation to use metformin as the first-line antidiabetic drug in many treatment guidelines remains reasonable in consideration of the multiple pleiotropic benefits including the prevention of GPD associated with its use.

## Data availability statement

The datasets presented in this article are not readily available because public availability of the dataset is restricted by local regulations to protect privacy. Requests to access the datasets should be directed to C-HT, ccktsh@ms6.hinet.net

## Ethics statement

This study was reviewed and approved by The Research Ethics Committee of the National Health Research Institutes. Written informed consent for participation was not required for this study in accordance with the national legislation and the institutional requirements.

## Author contributions

C-HT acquired funding, researched data, planned analyses, controlled quality and wrote manuscript. The author confirms being the sole contributor of this work and has approved it for publication.

## Funding

Financial supports have been provided for the study from the National Science Council (NSC 102-2314-B-002-067), the Ministry of Science and Technology (MOST 103-2314-B-002-187-MY3) and the Yee Fong Charity Foundation.

## Conflict of interest

The author declares that the research was conducted in the absence of any commercial or financial relationships that could be construed as a potential conflict of interest.

## Publisher’s note

All claims expressed in this article are solely those of the authors and do not necessarily represent those of their affiliated organizations, or those of the publisher, the editors and the reviewers. Any product that may be evaluated in this article, or claim that may be made by its manufacturer, is not guaranteed or endorsed by the publisher.
